# Recruitment and retention of human autologous CD34+ CD117+ CD133+ bone marrow stem cells to infarcted myocardium followed by directed vasculogenesis: Novel strategy for cardiac regeneration

**DOI:** 10.1186/2052-8426-1-4

**Published:** 2013-12-13

**Authors:** Marek Malecki, Chelsea Sabo, Emily Putzer, Chris Stampe, Afsoon Foorohar, Carol Quach, Michael Beauchaine, Xenia Tombokan, Mark Anderson

**Affiliations:** Phoenix Biomolecular Engineering Foundation, San Francisco, CA USA; NMRFM, National Institutes of Health, Madison, WI USA; University of Wisconsin, Madison, WI USA; University of Sheffield, Sheffield, EU UK; Latin American Youth Center, Washington, DC USA; University of Minnesota, Minneapolis, MN USA; Western University, Lebanon, OR USA; Western University, Pomona, CA USA; AXS Bruker, Madison, WI USA; BioSpin Bruker, The Woodlands, TX USA

**Keywords:** Myocardial infarction, Regenerative medicine, Bone marrow stem cells, Stem cell therapy, Vasculogenesis, Heterospecific tetravalent antibodies

## Abstract

**Background:**

Ongoing clinical trials, in regenerative therapy of patients suffering from myocardial infarctions, rely primarily upon administration of bone marrow stem cells to the infarcted zones. Unfortunately, low retention of these cells, to the therapeutic delivery sites, reduces effectiveness of this strategy; thus it has been identified as the most critical problem for advancement of cardiac regenerative medicine.

**Specific aims:**

The specific aim of this work was three-fold: (1) to isolate highly viable populations of human, autologous CD34+, CD117+, and CD133+ bone marrow stem cells; (2) to bioengineer heterospecific, tetravalent antibodies and to use them for recruiting of the stem cells to regenerated zones of infarcted myocardium; (3) to direct vasculogenesis of the retained stem cells with the defined factors.

**Patients methods:**

Cardiac tissue was biopsied from the hearts of the patients, who were receiving orthotopic heart transplants after multiple cardiac infarctions. This tissue was used to engineer fully human *in vitro* models of infarcted myocardium. Bone marrow was acquired from these patients. The marrow cells were sorted into populations of cells displaying CD34, CD117, and CD133. Heterospecific, tetravalent antibodies were bioengineered to bridge CD34, CD117, CD133 displayed on the stem cells with cardiac myosin of the infarcted myocardium. The sorted stem cells were administered to the infarcted myocardium in the *in vitro* models.

**Results:**

Administration of the bioengineered, heterospecific antibodies preceding administration of the stem cells greatly improved the stem cells’ recruitment and retention to the infarcted myocardium. Treatment of the retained stem cells with vascular endothelial growth factor and angiopoietin efficiently directed their differentiation into endothelial cells, which expressed vascular endothelial cadherin, platelet/endothelial cell adhesion molecule, claudin, and occludin, while forming tight and adherens junctions.

**Conclusions:**

This novel strategy improved retention of the patients’ autologous bone marrow cells to the infarcted myocardium followed by directed vasculogenesis. Therefore, it is worth pursuing it in support of the ongoing clinical trials of cardiac regenerative therapy.

## Background

Myocardial infarctions result from occlusion of cardiac arteries leading to cessation of the heart’s blood supply, what causes necrosis of the cardiac muscle [1–3]. Cardiac and bone marrow stem cells are mobilized as natural healing response to the infarction [4–8]. That response may be enhanced by stimulation with pharmaceuticals or transgenes [9–12]. Unfortunately, those remedies may not be sufficient. Hence, the rationale for stem cell therapy is to boost processes of cardiac regeneration by supplying stem cells, which have to differentiate into cardiac cells in order to replace necrotic cells and to restore lost cardiac functions [13–17].

For the ongoing stem cell therapy clinical trials of cardiac regeneration, bone marrow and heart are the primary sources of the stem cells. Cardiac stem cells are more advanced in specialization toward cardiomyocytes, but they require surgery for acquisition and time for *in vitro* expansion [18, 19]. On the other hand, bone marrow is easily aspirated and instantly ready for administration in GMP regimes [20–25]. However, reported outcomes of these trials are inconsistent. Interpretations of the results’ variability include, but are not limited to, differences in: cell isolation and propagation procedures, viability of cells in therapeutic batches, purity of the cell batches with undetermined numbers of apoptotic/necrotic cells, numbers of administered cells, ways of monitoring numbers of cells recruited and retained to the therapeutic targets, incompatibility of the human stem cell biomarkers with those of non-humans determined in pre-clinical experiments, routes of the cells’ delivery, heterogeneity of marrow cells’ populations, and administration of unfractionated *vs* selected cell populations. The clinical trials in cardiac regeneration, using bone marrow enriched with populations of cells displaying CD34, CD117, and CD133, have been reported as most successful [19, 22–27]. Those reports match laboratory research data, which highlight cell surface expression of these biomarkers on human endothelial or myocardial progenitors [28–34].

The main mechanisms contributing to the stem cell based cardiac regeneration include: paracrine stimulation, cell fusion, and trans-differentiation [35, 36]. Nevertheless, in all these scenarios, the stem cells have to be delivered and retained to the treated tissues in sufficient numbers to attain therapeutic effects. Unfortunately, within 2 weeks, only 3-6% of the stem cells administered by infusion, or 6-12% of those administered by intramyocardial injection, remain detected at the sites of therapeutic interventions [13, 14, 37, 38]. This problem dramatically reduces therapeutic efficacy. Therefore, improving retention of the administered stem cells to the sites of therapeutic interventions has been recognized, as the most critical problem to resolve for improving efficacy of stem cell therapy [13, 37, 38].

To be retained, migrating and administered stem cells require solid scaffolds, within infarcted zones, to anchor onto. Upon infarction, the myocardial sarcolemmas are damaged. Some of the sarcomeric molecules are very quickly released to blood circulation, e.g., troponin, or light chains of myosin. Measuring their levels helps us to determine magnitudes of infarctions. The other molecules remain strongly incorporated into the architecture of sarcomeres, e.g., myosin heavy chains. Importantly, cardiac myosin also retains its antigenicity. Therefore, labeling with anti-myosin antibodies, modified with radioactive or superparamagnetic biotags, helps us to determine location and extent of infarction with PET or MRI. Therefore, cardiac myosin heavy chains are the most specific and stable structures in the infarcted zones to anchor the stem cells onto.

Equally important requirement for successful stem cell therapy is administration of cell batches with exquisite purity and excellent viability [38, 39]. This can be accomplished by thorough depletion of necrotic and apoptotic cells [40], as well as definite enrichment of selected batches with the aid of bioengineered fluorescent antibodies for gentle isolation by fluorescent activated cell sorting (FACS) at low rates with reduced pressure or superparamagnetic antibodies for magnetic activated cell sorting (MACS) at low field gradient [41–46].

The specific aim of this work was three-fold: (1) to isolate highly viable populations of human, autologous CD34+, CD117+, and CD133+ bone marrow stem cells; (2) to bioengineer heterospecific, tetravalent antibodies and to use them for recruiting of the stem cells to regenerated zones of infarcted myocardium; (3) to direct vasculogenesis of the retained stem cells with the defined factors.

## Methods

### Concept of novel strategy for cardiac regeneration with human autologous bone marrow cells

**Figure 1:**
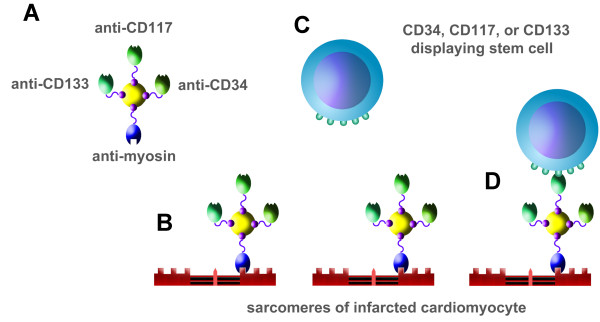
**Concept of novel strategy for cardiac regeneration with human autologous bone marrow cells. (A)** Heterospecific, tetravalent antibodies (htAbs) contain binding domains for four different antigens: CD34, CD117, CD133, and myosin. They are injected into the solution flowing over the sarcomeres of the infarcted myocardium. **(B)** The htAbs dock onto myosins of the sarcomeres. Excess of the htAbs is cleared from the circulation. **(C)** Human, autologous, bone marrow stem cells (haBMSCs) are spiked into the solution. **(D)** The cells displaying on their surfaces CD34, CD117, CD133 are recruited and retained to the infarcted myocardium sarcomeres with the aid of the htAbs (only CD117 cell is shown here for clarity). The retained haBMSCs are directed to differentiate into endothelium with the defined factors (not shown).

Novel strategy for recruitment and retention of the human, autologous, bone marrow stem cells (haBMSC) to the sarcomeres of the infarcted myocardia with the aid of the heterospecific, tetravalent antibodies (htAbs) is illustrated (Figure 1).

The foundation for this strategy was bioengineering of the htAbs. For this purpose, the monovalent nano-antibodies, each targeting CD34, CD117, CD133, and MHC, were modified to carry the single biotin group at their carboxyl termini. One at a time, these antibodies were docked into avidin, thus forming sequentially mono-, bi-, tri-, or tetra-valent antibodies. Since each of the four, incorporated, monovalent nano-antibodies was different, then the final antibody was the htAb. Therefore, these htAbs worked as the bridges between myosin in sarcomeres of the infarcted myocardium and CD34, CD117, CD133 displayed on surfaces of the administered human, autologous bone marrow stem cells (haBMSCs).

Five steps were involved in pursuit of this strategy: (A) administration of htAbs; (B) recruitment of the htAbs onto the myocardial sarcomeric myosin; (C) administration of haBMSCs; (D) recruitment of the haBMSCs onto the anchored htAbs; (E) directed differentiation of the recruited haBMSCs into endothelium. *In vitro* simulation of this therapeutic strategy is described below.

### Patients, cardiac tissue, bone marrow

All the samples were acquired in accordance with the Declaration of Helsinki with the Patients’ Informed Consent and with the Institutional Review Boards’ approval.

Bone marrow aspirates were acquired from six patients receiving orthotopic heart transplants. The surgical procedures were performed in the sterile conditions after induction of general anesthesia. Using heparinized, sterile needles, approximately 10 ml volumes of bone marrow were aspirated from the iliac crests. No iatrogenic complications were ever reported.

Populations of the desired cells were isolated directly from the bone marrow aspirates. They were labeled with the bioengineered antibodies targeting CD34, CD117, and CD133 as described earlier [34, 40]. The antibodies were rendered fluorescent or superparamagnetic [40]. These antibodies were applied at the concentration of 0.01 pg of antibody/1 × 10^4 cells for 30 min at 4°C on ice in darkness, while rocked on gyroscopic tables. The isotype nano-antibodies were used as the controls. The fluorescent or superparamagnetic antibodies, against double stranded DNA and phosphatidylserine, were used to remove necrotic or apoptotic (respectively) cells. The yields varied, but the cell numbers were reaching 1 × 10^4 – 2 × 10^10^6 per sample. Three rounds of isolations were pursued. The isolated cells were further analyzed by flow cytometry or lysed for the native receptors immuno-magnetic precipitation, electrophoresis, and blotting as described [40].

The cardiac tissues were obtained from the hearts of the recipients of heart transplants in orthotopic procedures. Immediately after the hearts were released during the open chest surgery, they were immersed into the ice-cold University of Wisconsin solution and the samples were excised from the central zone of the infarcted myocardium. Thereafter, the cardiac samples were prepared as: (1) myofibrils; (2) sections; (3) cryo-mounts; (4) primary cultures; (5) homogenates.

Strips of the cardiac muscle tissue were brought to a stretched or contracted state and clamped with the U shaped vascular surgery forceps. They were immersed in the solution (75 mM KCI, 10 mM Tris pH6.8, 2 mM EGTA, 2 mM MgCl2, 0.1 mM PMSF, 0.1% Triton X-100). The tissues were homogenized in a Polytron (Brinkman Instruments Co., Westbury, NY, USA) and a Teflon glass homogenizer. Myofibrils were collected by centrifugation at 1,000 g for 5 min. The pellets were washed by cycles of re-suspension and centrifugation. Finally, they were infused with the fresh buffer containing 50% glycerol and frozen at -20°C for storage. They were thawed and rinsed with the fresh buffer before use.

Cardiac tissues were rapidly cryoimmobilized in the HPM 010 (Balzers, Lichtenstein, EU). The frozen muscles were either sectioned in the frozen hydrated state or cryo-substituted, infused with 2.3 M sucrose, refrozen, and sectioned on the cryoultramicrotome (Leica, Vienna, A, EU). Alternatively, the frozen tissues were crushed for homogenates.

Small cubes of the fresh cardiac tissues were disintegrated with the sterile, surgical scalpel and plated onto the Petri dishes with the bottoms covered by matrigel or native cardiac tissue sections and filled with the DMEM supplemented with serum, powdered cardiac tissues, and antibiotics. The primary cultures were grown in the incubators maintaining 37°C, 10% CO_2_, and saturated humidity. For storage, the tissue cultures were infused with DMSO or glycerol and frozen gradually to retain their viability.

The cryoimmobilized samples were crushed, frozen, and lyophilized. They were used after rehydration as the media supplement or studied by electrophoresis and blotting.

All these approaches assured preservation of the native state of the cardiac muscle protein antigenicity and architecture. All specimens were examined by flow cytometry (FCM), multiphoton fluorescence spectroscopy (MFS), nuclear magnetic resonance spectroscopy (NMRS), energy dispersive x-ray spectroscopy (EDXS) [38, 45].

### Bioengineering of heterospecific, tetravalent antibodies

Heterospecific, tetravalent antibodies were bioengineered as described [45]. Briefly, the B cells were isolated from the blood of patients suffering cancers and myocardial infarctions. The pooled B cells from these patients were used to isolate mRNA, which was reverse transcribed to create the human cDNA libraries. The cds, after insertion into the plasmids containing chelates’ harboring coding sequences under the CMV promoters and terminated with polyA, were propagated and expressed in human myelomas or B cells. Gene shuffling enhanced the libraries’ diversities. The native CD34, CD117, CD133 were purified by immunoprecipitation with antibodies, which followed by their modification with biotin, digoxigenin, or fluorescein. They were anchored onto anti-biotin, anti- digoxigenin, or anti- fluorescein saturated pans and served as baits for selection of the expression libraries. The chelates were saturated with Gd, Tb, Ru, and Eu. The specificity and sensitivity were determined based upon elemental compositions with EDXS (Noran, Middleton, WI, USA), EELS (Zeiss, Oberkochen, D, EU), or TRXFS (Bruker AXS, Fitchburg, WI*,* USA). The fluorescent properties were measured with the RF-5301PC spectrofluorometer (Shimadzu, Tokyo, Japan). The magnetic relaxivities were measured on the DMX 400 WB or AVANCE II NMR spectrometers (Bruker Optics, Dallas, TX, USA).

For preparing tetravalent antibodies, the first batch of the bioengineered, monovalent antibodies was sprayed from an air-brush with a single pulse over the pan filled with the 0.001 mg/mL recombinant avidin (rA) in PIPES buffer in a saturated humidity chamber maintained at room temperature. Upon complete binding, the fractions of resulting solution were separated by the size exclusion chromatography on the high pressure liquid chromatography (HPLC) (Pharmacia, S, EU) columns. The fractions were collected on the fraction collector (Pharmacia, S, EU). The fractions detected to contain rA linked with the single monovalent antibody were pooled together and sprayed over the new pan. The procedure was repeated for all the antibodies, one at a time, in a random order. The system was calibrated using peaks for classic IgG, Fab, Fc, and avidin, as the references.

### Fluorescent, activated cell sorting. Flow cytometry. Multiphoton fluorescence spectroscopy

The marrow cells were labeled with the fluorescent, bioengineered antibodies targeting CD34, CD117, and CD133 and for negative selection with the antibodies targeting double stranded DNA (dsDNA) and phosphatidylserine (PS) in a single step at 4°C in darkness for 30 minutes [40]. They were sorted on the Calibur, Vantage SE, or Aria (Becton-Dickinson, Franklin Lakes, NJ, USA). The antibodies were dissolved and all washing steps carried in phenol-free, Ca^+^/Mg^+^- free, PIPES buffered saline solution, supplemented with 20 mM glucose, 5% human serum. The labeled marrow cells were sorted on Aria, Calibur, Vantage SE (Becton-Dickinson, Franklin Lakes, NJ, USA) with the sheath pressure set at 20 pounds per square inch pressure and low count rate. The sorted batches were analyzed on Calibur or Aria using FACSDiva software or on the FC500 (Beckman-Coulter, Brea, CA, USA). For the measurement of the fluorescently labeled cells, these settings were tuned at the maximum emission for the Eu chelated antibody at 500 V with references to isotype antibodies and non-labeled cells. This assured the comparisons between populations of cells labeled with multiple antibodies without changing the settings on PMTs.

The fluorescently labeled cells or tissues were imaged with the Axiovert (Zeiss, Oberkochen, D, EU) equipped with the Enterprise argon ion (457 nm, 488 nm, 529 nm lines) and ultraviolet (UV) (364 nm line) lasers; Odyssey XL digital high-sensitivity with instant deconvolution confocal laser scanning imaging system operated up to 240 frames*/*s (Noran, Madison, WI, USA), and the Diaphot (Nikon, Tokyo, Japan) equipped with the Microlase diode-pumped Nd:YLF solid state laser (1048 nm line) (the multi-photon fluorescence station built based upon the NIH funds – Principal Investigator: Dr J. White).

### Nuclear magnetic resonance spectroscopy. Magnetic activated cell sorting

The marrow cells were labeled for positive selection with the superparamagnetic nano-antibodies targeting CD34, CD117, CD133, and for negative selection with the antibodies targeting double stranded DNA (dsDNA) and phosphatidylserine (PS) [40]. The antibodies were dissolved and all washing steps carried in phenol-free, Ca^+^/Mg^+^- free, PIPES buffered saline solution, supplemented with 20 mM glucose, 5% human serum. The aliquots were dispensed into the magnetism-free NMR tubes (Shigemi, Tokyo, Japan). The relaxation times T1 were measured in resonance to the applied pulse sequences on the NMR spectrometers: DMX 400 WB or AVANCE II NMR (Bruker, Billerica, MA*)* or the Signa clinical scanners (GE, Milwaukee, WI, USA).

The superparamagnetic nano-antibodies were also used to isolate the labeled cells from the solution. The marrow cells labeled with the superparamagnetic antibodies were isolated on the magnetic, activated cell sorter operated at 1.5 T (the superparamagnetic bioengineered antibodies and sorter designed and built based upon the NSF funds – Principal Investigator: Dr M. Malecki).

### Energy dispersive X-ray spectroscopy. X-ray reflection fluorescence spectroscopy

The samples, which were cryo-immobilized, presented the life-like antigenicity and supramolecular organization. Elemental analyses were pursued by EDXS and XRFS as described [40]. The field emission, scanning transmission, electron microscope FESTEM HB501 (Vacuum Generators, Kirkland, WA, USA) was equipped with the energy dispersive x-ray spectrometer (EDXS) (Noran, Middleton, WI, USA) and post-column electron energy loss spectrometer (EELS) (Gatan, Pleasanton, CA). The cryo-energy filtering transmission electron microscope 912 Omega was equipped with the in-column, electron energy loss spectrometer (EELS) and the energy dispersive x-ray spectrometer (EDXS) (Zeiss, Oberkochen, D, EU). The cryo-energy filtering transmission electron microscopes 410 and 430 Phillips were equipped with the post-column, electron energy loss spectrometers (EELS) and the energy dispersive x-ray spectrometer (EDXS) (Noran, Middleton, WI, USA). The field emission, scanning electron microscope SEM1530 (Zeiss, Oberkochen, D, EU) was equipped with the energy dispersive x-ray spectrometer (EDXS) (Noran, Middleton, WI, USA). The field emission, scanning electron microscope 3400 was equipped with the energy dispersive x-ray spectrometer (EDXS) (Hitachi, Tokyo, Japan). The S2 Picofox XRFS spectrometer was equipped with a molybdenum (Mo) X-ray target and the Peltier cooled Xflash Silicon Drift Detector (Bruker AXS, Fitchburg, WI, USA). Scan times ranged up to 1000 seconds. The ICP standard of 1000 mg/l of mono-element Gallium or Gadolinium (CPI International, Denver, CO, USA) was added to 500 microL of each sample to the final concentration of 10 mg/l. Instrument control, data collection, and analysis were under the SPECTRA 7 software (Bruker AXS, Fitchburg, WI, USA).

### Quantitative reverse transcription and polymerase chain reaction

Total RNA was isolated with TRIzol (MRC, Cincinnati, OH, USA). RNA served as the template to generate cDNA through reverse transcription using random hexamers and reverse transcriptase (ABI, Foster City, CA, USA). The transcripts for GAPDH and actin served as the controls (ABI, Foster City, CA, USA). They were synthesized on the 380A DNA Synthesizer (ABI, Foster City, CA, USA). The PCR reactions were carried using the mix of the cDNA, the synthesized primers, dNTPs, and Taq DNA polymerase (Hoffmann–La Roche, Basel, H) on the Robocycler (Stratagene, San Diego, CA, USA), Mastercycler (Eppendorf, Hamburg, D, EU), and 7500 or 7900 systems (ABI, Foster City, CA, USA). The images of the electrophoresed amplicons were acquired and quantified with Fluoroimager (Molecular Dynamics, Sunnyvale, CA, USA) or Storm 840 (Amersham, Buckinghamshire, UK, EU). The levels of the transcripts were all normalized against GAPDH or actin. Thereafter, they were calculated as the ratios between the transcripts’ concentration in the examined patient’s cells versus the cells from the healthy control tissues and cultures.

### Immunoblotting

The cells and tissues were either frozen and crushed or disintegrated with ultrasonicator (Branson Ultrasonic, Danbury, CT, USA) and homogenized within the sample buffer. They were stored in liquid nitrogen. They were electrophoresed in the native buffer (Invitrogen, Carlsbad, CA, USA). They were vacuum- or electro-transferred onto the PVDF membranes (Amersham, Buckinghamshire, UK, EU). The membranes carrying the transferred proteins were first soaked within human serum and thereafter labeled with the bioengineered antibodies. The purified CD34, CD117, CD133, and cardiac muscle myosin served as the controls. The images of the blots were acquired and quantified with Fluoroimager (Molecular Dynamics, Sunnyvale, CA, USA) or Storm 840 (Amersham, Buckinghamshire, UK, EU).

### Targeting and retention of the human, bone marrow stem cells

The chambers were filled with cardiac tissues and tightly sealed. Solutions were propelled to flow through the chambers by the peristaltic pump (Flowrox, Linthicum, MD, USA). The chambers were connected with the environmental incubator through flexible Tygon hoses. That assured maintaining of the sarcomeres and the bone marrow cells at 37°C, pH 7.3, 120/80 mmHg, and 330 mOsm. Cardiac α-actinin of sarcomeres was labeled with antibodies modified with Gd or FITC as the internal references. The cells were tagged with fluorescent of superparamagnetic or fluorescent antibodies. Under the continuous flow, the bone marrow stem cells were administered. At various time intervals, the flow was stopped and the number of the retained stem cells quantified based upon the changes in ratios of fluorescence or relaxivity.

### Directing vasculogenesis of the stem cells retained to sarcomeres

Upon completion of the recruitment of the bone marrow stem cells to the cardiac tissues, the solution, flowing through the chambers, was Vascular Cell Basal Medium (ATCC, Arlington, VA, USA) supplemented with recombinant human Vascular Endothelial Growth Factor 50 ng/mL, recombinant human Epidermal Growth Factor 5 ng/mL, recombinant human Basic Fibroblast Growth Factor 5 ng/mL, recombinant human Insulin-like Growth Factor 15 ng/mL, angiopoietin-1 20 ng/mL, L-glutamine 10 mM, heparin sulfate 0.75 Units/mL, hydrocortisone hemisuccinate 1 μg/mL, ascorbic acid 50 μg/mL. At various time intervals, endothelial differentiation was validated by monitoring expression of the uniquely specific genes: *TJP1* for zona occludens; *OCLN5* for endothelial occludin; *CLDN5* for endothelial claudin*; PECAM1* for platelet/endothelial cell adhesion molecule 1; *CTNNB* for catenin cadherin-associated protein; and *CDH5* for vascular endothelium cadherin 5. For imaging, fluorometry, and flow cytometry, these genes were expressed as fusions with green fluorescent protein (GFP) and its mutations blue, yellow, cyan, orange, and red and gene expression products were labeled with nano-antibodies [40–46]. Human Normal Primary Artery Endothelial Cells served as the positive and Human Bone Marrow served as the negative controls (ATCC, Arlington, VA, USA). Imaging, blotting, and amplification were pursued as outlined below.

### Statistical analysis

All the measurements were run in triplicates for each sample from six patients (three women and three men). The numbers were analyzed and displayed using GraphPad software (GraphPad Software, Inc, La Jolla, CA). Data were presented as mean ± standard error of the mean (SEM). Statistical significance was calculated by t-test for two groups.

## Results

**Figure 2:**
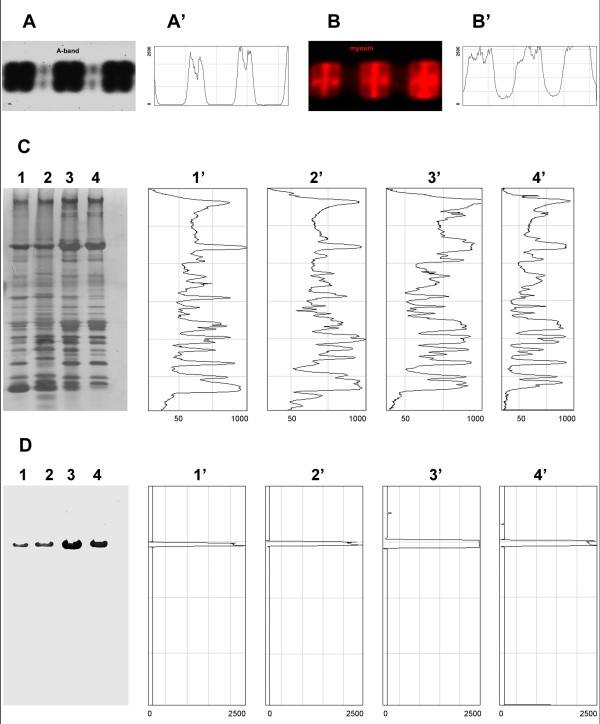
**Preservation of architecture and antigenicity of myosin in infarcted myocardia. (A)** The Zernicke’s phase-contrast microscopy showed the overall structure of the sarcomeres in the human, native, cardiac myofibrils from the central zone of infarcted myocardium. **(B)** Cardiac myosin (in the sarcomeres of the myofibrils from **A)** was labeled with the fluorescent htAbs against myosin and highlighted in multiphoton fluorescence spectroscopy. The anti-myosin htAbs overlap exactly the A-bands of the sarcomeres. **(C)** Gels of the four patients' (MI/HT001-004) cardiac tissues, which were disintegrated, electrophoresed, and stained, revealed prominent and sharp bands of myosin, actin, tropomyosin, troponin, actinin, and titin. These show preservation of molecular integrity of the sarcomeric proteins. **(D)** The same samples as those shown in **C**, were transferred onto PVDF membranes, and labeled with the htAbs against myosin. The only bands are exquisitely specific for the labeled myosin. These are also indications of preservation of myosin antigenicity. The entire lanes are shown to demonstrate very specific labeling and lack of any non-specific binding. Axial pixel brightness compared between specific signals and backgrounds was accepted at *P* = .0003.

To assess preservation of the architecture and antigenicity of myosin in infarcted myocardia, which were both critical for anchoring human autologous bone marrow stem cells (haBMSCs), the sarcomeres were studied by multiphoton fluorescence spectroscopy and immunoblotting, while the results are illustrated (Figure 2).

The overall structure of the native myofibril sarcomeres is shown in the Zernicke’s phase contrast. A-bands and Z-lines are well preserved with sharp edges and no deterioration of architecture. The A-bands constitute majority of the sarcomeres’ volume; thus the largest binding surface for antibodies intended to dock onto sarcomeres. Projection of the myosin labeling pattern onto the myofibrils shows exact overlapping with the A-band. There is absence of non-specific labeling in the background. All the samples were run in triplicates. The images of sarcomeres from the tissue biopsied from this patient’s heart were representative for all samples studied. It is indicative of myosin’s preserved antigenicity, which is the critical feature for specific binding of the htAbs.

Preservation of sarcomeric proteins was demonstrated by electrophoresis. It revealed sharp bands in the classical pattern of cardiac muscle proteins in all the patients’ samples. Prominent bands of myosin, as well as bands of actin, actinin, and tropomyosin are clearly distinguished. The myosin labeling patterns after electro-transfer and immunoblotting can be projected onto the lanes of the electrophoresed cardiac muscle. The bands corresponding to myosin are heavily labeled with the htAbs. All the samples were run in triplicates. The myosin blot pattern revealed on the biopsy from these patients were representative for all samples studied. The labeling is very specific, which is critical for using the htAbs for recruiting the haBMSCs.

**Figure 3:**
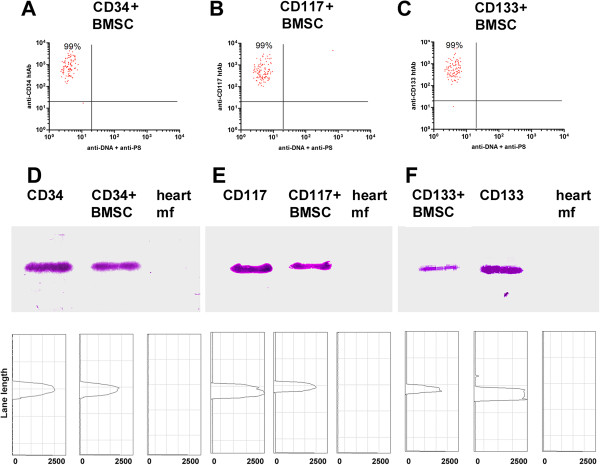
**Purity and viability of isolated, human, autologous, bone marrow stem cells (haBMSCs). (A-C)** Batch purity after labeling with the superparamagnetic htAbs targeting CD34 **(A)**, CD117 **(B)**, CD133 **(C)** and sorting by MACS was assessed by flow cytometry. Dead cells – labeled with antibodies against double stranded-DNA (anti-dsDNA) and apoptotic – labeled with antibodies against phosphatidylserine (anti-PS) cells were sorted out entirely so are not detected by flow cytometry (empty Q4). The counts were averaged and normalized against first 1000 events. **(D-F)** Batch purity was further assessed on immunoblots. Isolated populations of haBMSCs (BMSC CD34, BMSC CD117, BMSC CD133) and heart tissues (heart mf) were homogenized and lysed. They were electrophoresed on parallel lanes to purified clusters of differentiation (CD34, CD117, CD133). All electrophoresed samples were transferred onto PVDF membranes and labeled with the htAbs targeting CD34 **(D)**, CD117 **(E)**, CD133 **(F)**. The immunoblots revealed presence of the only cells displaying the targeted receptors. Since there were no other bands on the blots, then there was no false positive labeling of other receptors, thus no falsely positive cells in these batches.

To test purity and viability of isolated populations of bone marrow cells, they were analyzed by flow cytometry (Figure 3). The batches of these cells featured exquisite purity. Negative selection with the antibodies against double stranded DNA (anti-dsDNA) and phosphatidylserine (anti-PS) ensured absence of dead and apoptotic/dying cells; thus assured high viability of the haBMSCs planned for being used for stem cell therapy. All the samples were run in triplicates and shown patterns were representative for all studied.

To validate purity of the isolated batches of stem cells after rounds of sorting, the batches of stem cells were tested by electrophoresis and immunoblotting. Purified CD34, CD117, CD133 were used as the positive controls. All the samples were run in triplicates. The immunoblotting patterns revealed on these samples were representative for all studied. The labeling is uniquely specific for the CD34, CD117, CD133, as validated by the identical bands of labeling on the lanes carrying lysates of the batches of the haBMSCs, as on the lanes carrying only the purified receptors as the specificity controls. There were neither other cells detected, nor any other molecules labeled, but the ones specifically displaying biomarkers targeted by the htAbs.

**Figure 4:**
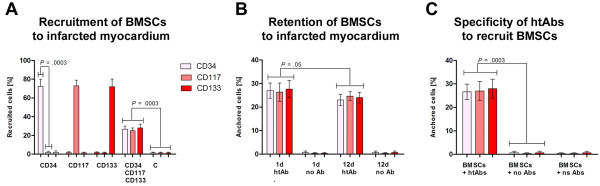
**Recruitment and retention of haBMSCs to infarcted myocardia. (A)** The isolated populations of cells with the cell surface display of CD34, CD117, CD133 (each tagged with a different elemental tag) were applied either as a single batch of haBMSCs mixed with clusters of differentiation saturating remaining binding sites (e.g., haBMSCs CD34+ with CD117, CD133) or as a mix of all three populations in equal ratios. They were compared to the assay in which the haBMSCs were applied without preceding htAbs (“C” in this figure). Applying all three native receptors blocked binding sites on the htAbs and prevented the haBMSCs from docking. The statistical significance was accepted at *P* = .0003. **(B)** Retention of the haBMSCs onto the sarcomeres was measured at different periods of time for samples maintained in the environmental incubator. The difference between the numbers of cells retained to the sarcomeres, when they were preceded by administration of the htAbs (htAb) was much higher than preceded by administration of plain buffer with no antibodies (no Ab). Once the haBMSCs anchored to the sarcomeres on the day one of the experiment (1d) , thereafter they were retained with minimal losses for up to 12 days studied (12d). The statistical significance was accepted at *P* = .0003. **(C)** Specificity of recruitment of the CD34+, CD117+, CD133+ haBMSCs’ populations was determined after mixing them in equal ratios and administration to myocardial infarction models, while preceded by injections of the htAbs (htAb), non-specific antibodies (nsAbs), or plain buffer without any antibodies (no Ab). In the assays, which included htAbs, the numbers of the cells attached were much higher, totaling more than 80%, in comparison with the assays including non-specific or excluding all antibodies. The statistically significant difference was accepted at *P* = .0003.

Impact of the htAbs, upon recruitment and retention of the haBMSCs to the infarcted myocardial sarcomeres, is summarized (Figure 4). In all the assays, injections of the haBMSCs were preceded by injections of: either the htAbs, or blocking antibodies, or blocking ligands, or non-specific antibodies, or plain buffers with no antibodies. Quantification was performed with the energy dispersive x-ray spectroscopy (EDXS), x-ray fluorescence spectroscopy (XRFS), or nuclear magnetic resonance spectroscopy (NMRS). Permanent labeling of the haBMSCs with elemental biotags facilitated measurements of emitted x-ray radiation by EDXS and XRFS, which was proportional to the number of cells retained. Labeling with superparamagnetic biotags promoted measurements of relaxivities by NMRS. The quantification was normalized against the total myocardial tissue mass.

Efficacy of the htAb-aided recruitment was quantified either for the total number of bone marrow cells mixed together or separately for populations of the isolated CD34+, CD117+, and CD133+ cell batches. Total percentages of the anchored cells were exceeding 80%. In the mixtures of three cell populations, the ratios between the cells anchored were approximately the same as the ratios between the cells administered. The data collected in triplicates for each patient are presented as cumulative for all the patients as mean with standard deviations. These measurements revealed the statistically significant improvement in recruitment of the haBMSCs with the aid of the htAbs as compared to the assays without the htAbs or non-specific Abs.

Binding was very specific, as demonstrated by the counts of the cells attached to the sarcomeres, when the binding sites for the htAbs were selectively blocked either on the sarcomeres with anti-myosin antibodies or on the haBMSCs with anti-CD34, CD117, and CD133 antibodies. Selective blocking of the binding sites on the htAbs with the ligands resulted in the reduced recruitment of the corresponding haBMSCs’ fractions.

Retention of the recruited haBMSCs was measured at different time intervals. Only minimal losses of the anchored cells were measured in experiments lasting for up to two weeks. During those periods of time, the cells demonstrated high viability of the retained cells, as determined by labeling with the superparamagnetic or element tagged antibodies against double stranded DNA (anti-dsDNA) and phosphatidylserine (anti-PS).

Ultimately, the results of this work were intended to enhance efficacy of cardiac regeneration therapy by directed neo-vascularization of the infarcted zones. Therefore, the primary task for this project was to stimulate vasculogenesis of the haBMSCs anchored to myocardia. This was accomplished by treating the haBMSCs retained to the sarcomeres with the vascular endothelial growth factor (VEGF) and angipoietin-1 (Ang-1). These factors efficiently triggered expression of genes unique for angiogenesis.

**Figure 5:**
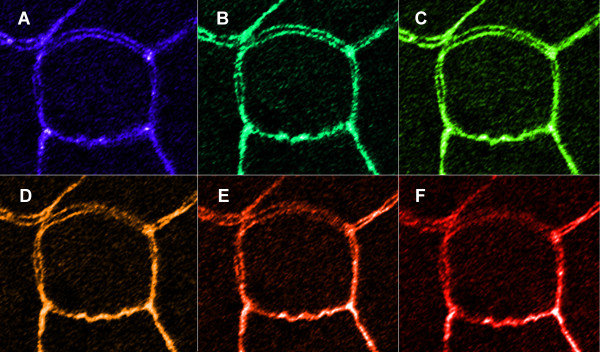
**Directed vasculogenesis of haBMSCs retained to infarcted myocardium.** Vasculogenesis was assessed by monitoring expression of the unique genes leading to assembly of tight junctions **(A-C)** and adherens junctions **(D-F)**. These phenomena were highlighted by transgenic expression of fluorescent-junction fusion proteins and fluorescent nano-antibody labeling of junction proteins (intermittent with photobleaching), while images were acquired on high-speed, high sensitivity confocal system and followed by instant deconvolution to eliminate spectral overlaps and resonance energy transfers, as described and validated [40, 45]. Labels are: occludin (**A** - blue), claudin (**B** - cyan), zona occludens (**C**- green), VE cadherin (**D** - yellow), catenin (**E** – orange), platelet/endothelial cell adhesion molecule (**E** – red). The colors are assigned artificially, but correspond to wave-lengths of the narrow band-width optical filters. Unique “cobble stone” geometry of the haBMSCs directly differentiated into endothelium is revealed.

To assess the functional features of human bone marrow cells, which were directed to differentiate into endothelium, while discriminating between them and cells already present in the myocardial tissue, transgenic expression of fusion proteins as reporters was studied (Figure 5). Functionality of endothelium is contingent upon formation of intercellular junctions. Therefore, this work was focused on studies of gene expression products - proteins, which are responsible for forming tight junctions: occludin and claudin, as well as adherens junctions: vascular endothelial cadherin and platelet/endothelium adhesion molecules. These proteins were imaged with the high speed, high sensitivity, high spectral resolution confocal system, which was capable to detect low intensity fluorescence and to discriminate small wave-length shifts, while compensating for possible frictions of cardiac tissues and administered cells during acquisitions of images. For each patient, the images were acquired in triplicates and the ones presented are representative to all. The tight junctions and adherens junctions were effectively formed, while highlighted.

## Discussion

The results of this work constitute the proof of concept, in the fully human *in vitro* model, for resolving the most critical problem in regenerative medicine of the myocardial infarctions: recruitment and retention of the stem cells to the sites of therapeutic interventions [13]. Herein, we describe resolution of this problem by bioengineering of heterospecific, tetravalent antibodies (htAbs) and using them for recruitment and retention of selected populations of bone marrow stem cells to infarcted myocardium.

We attribute high efficacy of the htAbs, in anchoring of the bone marrow stem cells to the human cardiac infarcted muscle sarcomeres, to several factors. (1) The htAbs have exquisite specificity and affinity towards cardiac myosin. Therefore, their exclusive targets are molecules of myosin, which are present only in the regions of the damaged cardiomyocytes. (2) Myosin retains its antigenicity and accessibility to serve as a solid anchoring scaffold. (3) The htAbs have high specificity towards CD34+, CD117+, and CD133+ stem cells. Therefore, pure batches of stem cells are obtained and presented to the infarcted myocardium. (4) The anti-dsDNA and anti-PS are effective in eliminating all dead and dying cells. Therefore, only viable cells, with high differentiation potential, are administered. (5) The model of therapy includes all the human-specific components taken directly from the injured heart. Therefore, they are thoroughly tested in the environment *in vitro,* which is closest to the conditions *in vivo*.

Numbers of recruited cells may be reduced with time due to dying or migration. The conditions in the *in vitro* model of myocardial infarction included the patients’ infarcted tissue and serum; thus relatively thorough simulation of the *in vivo* environment. The number of retained and surviving cells was very high in this study. Nevertheless, only *in vivo* trials, with active reticulo-endothelial and immune systems, will provide validation of this strategy *in vivo* in long term clinical trials.

The presented strategy consists of two elements: human autologous bone marrow and htAbs. Using haBMSCs reduces the problems associated with the immune response, as well as with the iatrogenic injuries associated with introducing immuno-suppression. However, the htAbs may result in the immune response after multiple applications, if they are not produced by the patients’ own B cells. We are vigorously working on resolving this problem.

We are also aware that the isolated cells may co-display many other biomarkers, as a reflection of their differentiation stages. As such, they may be featuring spectra of regenerative potentials. Therefore, in the next task, we are trying to identify most suitable sub-populations. We are trying to accomplish this by multi-parameter, magnetic or fluorescent sorting with the aid of the new, bioengineered htAbs.

The results of this work also constitute the proof of concept *in vitro* for directed differentiation, of the selected populations of the human autologous bone marrow stem cells into endothelium in situ - at the site of therapeutic intervention.

These results are well aligned with other approaches to generate endothelial progenitors from human induced pluripotent stem cells and human embryonic stem cells, which followed by advancing their differentiation into endothelial cells, formation of tight junctions, migration into the neighboring areas to reach other cell clusters, and assembling into networks of endothelial cells. However, one of the tests of pluripotency of induced and embryonic stem cells is their ability to form teratomas *in vivo*[47–50]. This carries the risk of neoplasmic transformation, when streamlined into the clinical setting. Although, methods to safeguard therapeutic use of pluripotent stem cells are being developed, the strategy proposed herein offers an alternative worth pursuing.

Finally, if this strategy would be considered to become a part of clinical trials, then all of the components would have to be non-toxic for humans, have long shelf-life, have controlled pharmacokinetics, and be manageable in GMP environment. All factors introduced in this project meet these requirements. They exerted their action, while in the totally controlled environment, which contained all the same human molecules and cells, which they would be interacting with, in the *in vivo* first-in-man trials.

## Conclusions

This novel strategy improved retention of the patients’ own bone marrow cells to the infarcted myocardia followed by directed vasculogenesis. Therefore, it is worth pursuing it in support of the ongoing clinical trials of cardiac regenerative medicine.

## Abbreviations

BMSC: Bone marrow stem cell
heSCs: Human embryonic stem cells
htAb: Heterospecific tetravalent antibody
mAb: Monoclonal antibody
Fab: Antigen binding fragment antibody
scFv: Single chain variable fragment antibody
SSEA-4: Stage specific embryonal antigen 4
SSEA-3: Stage specific embryonal antigen 4
TRA-1-60: Tumor related antigen recognized by the monoclonal antibody 1–60
TRA-1-81: Tumor related antigen recognized by the monoclonal antibody 1–81
FCM: Flow cytometry
IB: Immunoblotting
MACS: Magnetic activated cell sorting
FACS: Fluorescent activated cell sorting
NMRS: Nuclear magnetic resonance spectroscopy
XRFS: X-ray fluorescence spectroscopy
MPFS: Multiphoton fluorescence spectroscopy
EELS: Electron energy loss spectroscopy
EDXS: Energy dispersive x-ray spectroscopy.
